# Predictive Factors for Catecholamine-Induced Cardiomyopathy in Patients with Pheochromocytoma and Paraganglioma

**DOI:** 10.3389/fendo.2022.853878

**Published:** 2022-03-08

**Authors:** Yi Wang, Xuerong Yu, Yuguang Huang

**Affiliations:** Department of Anesthesiology, Peking Union Medical College Hospital, Beijing, China

**Keywords:** pheochromocytoma, paraganglioma, cardiomyopathy, takotsubo cardiomyopathy, catecholamine

## Abstract

**Objective:**

To investigate possible predictive factors of catecholamine-induced cardiomyopathy in pheochromocytoma and paraganglioma (CICMPP) patients.

**Methods:**

In all, 50 CICMPP patients and 152 pheochromocytoma and paraganglioma (PPGL) patients without CICMPP who were treated in our institution between August 2012 and April 2018 were included in this retrospective study to assess predictors of CICMPP.

**Results:**

Patients with CICMPP reported younger onset age, more clinical symptoms and signs, more family history of hypertension, and higher maximum systolic, diastolic, and mean BP and maximum HR. Medical evaluation also showed higher level of blood hematocrit, blood glucose, 24-h urine catecholamines, larger diameter of the tumor and more comorbidities, von Hippel-Lindau syndromes, and metastatic tumors in these patients. Multivariable analysis identified maximum resting HR over 115 beats/min (OR 10.05, 95% CI 3.71–27.20), maximum resting systolic BP over 180 mmHg (OR 7.17, 95% CI 2.22–23.23), blood glucose over 8.0 mmol/L (OR 6.52, 95% CI 2.25–18.86), more than 3 symptoms and signs (OR 6.05, 95% CI 1.86–19.64), and onset age under 40 years (OR 3.74, 95% CI 1.37–10.20) as independent predictors of CICMPP. Female sex (OR 5.06, 95% CI 1.19–21.54), complaint of chest pain (OR 5.84, 95% CI 1.27–26.90), and extra-adrenal tumor (OR 8.64, 95% CI 1.82–40.94) were independent predictors of Takotsubo cardiomyopathy in CICMPP.

**Conclusion:**

Maximum resting HR ≥115 beats/min, maximum resting systolic BP ≥180 mmHg, blood glucose ≥8.0 mmol/L, number of symptoms and signs ≥3, and onset age ≤40 years were found to be predictive factors for CICMPP.

## Introduction

Pheochromocytomas and paragangliomas (PPGLs) are catecholamine-secreting tumors arising from chromaffin cells in the adrenal medulla and sympathetic ganglia, respectively, causing severely dangerous hypertension and high mortality rates even when the tumors are benign (1, 2). PPGLs have an annual incidence of 3–8 cases per one million per year (3). One of the leading causes of this high mortality rates is challenging circulatory management in the perioperative period due to the excess amounts of norepinephrine, epinephrine, dopamine, or the combination of these substances secreted by PPGL (4, 5). These excess catecholamines cause various symptoms and signs including headache, diaphoresis, palpitation, and hypertension, and also lead to multiple organ damages including catecholamine-induced cardiomyopathy.

Catecholamine-induced cardiomyopathy in PPGL (CICMPP) is a severe cardiac complication of PPGL associated with even higher morbidity and mortality rates compared to PPGL patients without CICMPP, resulting in cardiogenic shock, heart failure, acute renal failure, and lethal arrhythmias (6, 7), and often requires a longer medical preparation period (8). One-fifth to more than one-third of PPGL patients may suffer from cardiovascular complications (3). Appropriate medical treatment can recover left ventricular systolic function before surgery in half of these patients (9). One subtype of CICMPP worth noticing is Takotsubo cardiomyopathy, with the character of transient regional systolic dysfunction of the left ventricle without angiographic evidence of obstructive coronary artery disease or acute plaque rupture (10). It has a distinct histologic presentation of contraction band necrosis and sympathetic cardiac nerve terminal disruption with norepinephrine seethe and spillover (11). Takotsubo cardiomyopathy has a higher rate of left ventricular recovery before surgery compared to other cardiomyopathies (69.4% vs. 40.8%) (9), and a higher rate of recurrence of 20% (11) if left untreated.

Identifying CICMPP is crucial and yet difficult in some situations, because retrospective studies reported that the prevalence of CICMPP in PPGL is 8%–11% (12, 13). Cardiac MRI in PPGL patients revealed 59% had non-ischemic cardiac scarring (14). Furthermore, myocarditis was present in the autopsy of 50%–60% PPGL patients (15). Thus, identifying the predictive factors of CICMPP is an important issue. Due to the rarity of the disease, most reports on CICMPP are case reports. Several studies have reviewed published cases regarding the prevalence, clinical manifestation, and prognosis of CICMPP, revealing higher complication rates and lower left ventricular function compared to primary cardiomyopathies (9, 13, 16). Zelinka et al. summarized the cardiovascular complications in PPGL patients but lacked significant difference due to limited sample size (17). There have also been two reports on echocardiographic and electrocardiographic characteristics in PPGL patients (12, 18). However, there has not been a study on the predictive factors of CICMPP. Therefore, in this retrospective study, we aim to identify the predictive factors of CICMPP patients and predictive factors for different subtypes of CICMPP.

## Patients and Methods

From August 2012 to April 2018, 1,399 patients diagnosed with PPGL were taken care of in Peking Union Medical College Hospital. CICMPP was diagnosed in 52 patients. In a 1:3 matching ratio, 156 patients diagnosed as pheochromocytoma, thoracic, or abdominal paraganglioma without CICMPP in the same period drawn randomly were reviewed. Patients with incomplete clinical data other than pathology reports were excluded from the analysis; 2 CICMPP and 4 PPGL patients were excluded. CICMPP is defined as (1) acute chest pain and/or cardiac failure requiring hospitalization (2); biochemical, ECG, and/or echocardiographic evidence of myocardial ischemia with left ventricular systolic dysfunction; and (3) absence of obstructive epicardial coronary artery disease (19, 20). Takotsubo cardiomyopathy is defined according to the Johns Hopkins criteria (21). This study was approved by the Ethics Committee and Institutional Review Board of Peking Union Medical College Hospital (No. S-K1887).

Patient age, disease course, clinical symptoms, family history, maximum resting BP and HR, blood hematocrit, blood glucose, 24-h urine catecholamines, tumor characteristics, comorbidities, American Society of Anesthesiologists physical status (ASA-PS), New York Heart Association (NYHA) classification, and clinical presentations of genetic syndromes *MEN* and *VHL* were assessed to evaluate the potential relationship of these factors and CICMPP. Receiver operating characteristic analysis was then carried out on all the continuous variables that showed significant differences between the two groups. Cutoff points were determined as the value with which the Youden index (sensitivity + specificity − 1) in predicting CICMPP peaked. Multi-collinearity analysis was carried out before logistic regression to exclude variables sets with potential collinearity. Collinearity is considered present if variance inflation factor is over 10. Due to the limitation of the sample size, 5–6 variables were entered into binary logistic regression to identify the independent predictive factors each time. The same was done to identify independent predictive factors for Takotsubo cardiomyopathy in CICMPP patients.

Student’s *t*-tests were used to compare continuous variables with normal distribution, and Mann–Whitney *U* tests were used to compare continuous variables with non-normal distribution. Chi-square tests were used to compare categorical variables between two groups. Receiver operating characteristic analysis was used to determine the cutoff points for continuous variables. Binary logistic regression was used to examine the association of potential predictive factors with CICMPP. Continuous variables with normal distribution were presented as mean ± standard deviation (SD), and those with non-normal distribution median ± interquartile range (IQR). Categorical variables were presented as number (percentage). The association with Takotsubo cardiomyopathy was evaluated in a similar way. Statistical significance was considered as *p* < 0.05. Statistical analyses were performed using IBM SPSS Statistics version26 (IBM Inc., Chicago, IL, USA).

## Results

In all, 202 patients were included in this analysis; the mean age, height, and weight were 44.5 years, 165.4 cm, and 64.0 kg, respectively; the median BMI was 23.93, and 107 (53.0%) were female patients. There was no significant difference between the two groups in these demographic features (**Table 1**). The daily dosage of phenoxybenzamine was 45.0 ± 33.0 mg and 29.2 ± 18.4 mg for PPGL patients with and without CICMPP (*p* = 0.002). In addition to alpha-blockers, metoprolol was taken by 37 (74.0%) CICMPP patients and 21 (13.8%) PPGL patients without CICMPP (*p* < 0.001). The daily dosage of metoprolol is 41.9 ± 41.6 mg and 34.2 ± 15.0 mg for patients with and without CICMPP, respectively (*p* = 0.420). Calcium channel blockers were taken by 16 (32.0%) patients with CICMPP and 16 (10.5%) without (*p* = 0.001). The average length of hospital stay is 37.0 ± 20.1 days and 28.4 ± 21.7 days for patients with and without CICMPP, respectively (*p* = 0.016).

**Table 1:** The patients’ demographic characteristics.

Variable	CICMPP (*n* = 50)	PPGL without CICMPP (*n* = 152)	*p*
Age, years, mean (SD)	41.0 (17.1)	45.6 (12.5)	0.087
Sex, *n* (%)			
Male	24 (48.0)	71 (47.0)	0.874
Female	26 (52.0)	81 (53.0)	
Height, cm, mean (SD)	165.7 (8.7)	165.6 (8.3)	0.509
Weight, kg, mean (SD)	62.0 (12.4)	64.6 (12.3)	0.185
BMI, median (IQR)	23.52 (16.14)	24.05 (12.95)	0.333
Paragangliomas, *n* (%)	4 (8.0)	28 (18.4)	0.126

CICMPP, catecholamine-induced cardiomyopathy in PPGL.

PPGL, pheochromocytoma and paraganglioma.

Patients with CICMPP reported younger onset age, more clinical symptoms and signs (headache, diaphoresis, palpitation, hypertension, hypotension, chest pain, dyspnea, impaired tip perfusion, syncope, and nausea and vomiting), more family history of hypertension, and higher maximum resting systolic, diastolic, and mean BP and maximum HR than without. Upon medical evaluation, higher level of blood hematocrit, blood glucose, 24-h urine catecholamines, and larger diameter of the tumor was detected in CICMPP patients. There were also more diagnoses of von Hippel-Lindau syndrome (VHL) in CICMPP patients. Whereas the location of the tumor, whether the tumor was multiple, or whether a diagnosis of multiple endocrine neoplasia (MEN) or other endocrine tumors was made did not seem to have an influence. CICMPP patients also suffer more from metastatic tumors and comorbidities (hyperglycemia, hyperlipidemia, renal damage, retinal damage, hepatic damage, and multiple organ dysfunction syndrome (MODS)) and were graded higher in ASA and NYHA classifications (**Table 2**).

**Table 2:** Clinical characteristics between PPGL patients with vs. without CICMPP.

Variable	CICMPP (*n* = 50)	PPGL without CICMPP (*n* = 152)	*p*	Variable	CICMPP (*n* = 50)	PPGL without CICMPP (*n* = 152)	*p*
Onset age, years, mean (SD)	36.9 (15.0)	41.7 (11.8)	**0.041**	Maximum HR, beats/min, mean (SD)	126.5 (32.7)	95.3 (18.7)	**<0.001**
Disease course, months, mean (SD)	51.4 (42.0)	95.3 (86.0)	0.195	Blood			
Symptoms and signs				Hematocrit, %, median (IQR)	43.10 (6.70)	41.86 (2.50)	**0.034**
Headache, *n* (%)	34 (68.0)	65 (42.8)	**0.002**	Glucose, mmol/L, median (IQR)	9.20 (4.40)	6.75 (2.20)	**<0.001**
Diaphoresis, *n* (%)	35 (70.0)	42 (27.6)	**0.005**	24h urine			
Palpitation, *n* (%)	32 (64.0)	58 (38.1)	**0.001**	Norepinephrine, mcg, median (IQR)	259.20 (773.93)	97.33 (1,321.80)	**<0.001**
Hypertension, *n* (%)	47 (94.0)	91 (59.9)	**<0.001**	Epinephrine, mcg, median (IQR)	5.40 (8.11)	3.95 (25.10)	**0.037**
Hypotension, *n* (%)	5 (10.0)	2 (1.3)	**0.011**	Dopamine, mcg, median (IQR)	333.26 (336.99)	269.95 (126.04)	**0.015**
Chest pain, *n* (%)	17 (34.0)	3 (2.0)	**<0.001**	Tumor			
Dyspnea, *n* (%)	16 (32.0)	0 (0.0)	**<0.001**	Adrenal, *n* (%)	33 (66.0)	111 (73.0)	0.341
Impaired tip perfusion, *n* (%)	12 (24.0)	16 (10.5)	**0.017**	Multiple, *n* (%)	7 (14.0)	16 (10.5)	0.502
Syncope, *n* (%)	7 (14.0)	3 (2.0)	**0.003**	Diameter, mm, median (IQR)	56.0 (29.0)	46.8 (29.3)	**0.005**
Nausea and vomiting, *n* (%)	24 (48.0)	21 (13.8)	**<0.001**	Metastatic, *n* (%)	8 (27.6)	5 (5.2)	**0.002**
Number of symptoms and signs, median (IQR)	4 (2)	2 (2)	**<0.001**	Comorbidities			
Classic triad, *n* (%)	19 (38.0)	24 (15.8)	**0.001**	Hyperglycemia, *n* (%)	33 (66.0)	42 (27.6)	**<0.001**
No symptom, *n* (%)	0 (0.0)	30 (19.7)	**0.001**	Hyperlipidemia, *n* (%)	13 (26.0)	17 (11.2)	**0.011**
Family history				Renal damage, *n* (%)	12 (24.0)	1 (0.6)	**<0.001**
Hypertension, *n* (%)	19 (38.0)	34 (22.4)	**0.029**	Retinal damage, *n* (%)	18 (36.0)	8 (5.3)	**<0.001**
Pheochromocytoma, *n* (%)	3 (6.0)	3 (2.0)	0.163	Hepatic damage, *n* (%)	3 (6.0)	0 (0.0)	**0.014**
MEN, *n* (%)	1 (2.0)	8 (5.3)	0.457	Gastrointestinal bleeding, *n* (%)	2 (4.0)	0 (0.0)	0.060
VHL, *n* (%)	3 (6.0)	1 (0.6)	**0.048**	Cerebral bleeding, *n* (%)	2 (4.0)	1 (0.6)	0.152
Other endocrine tumors, *n* (%)	16 (32.0)	31 (20.4)	0.092	MODS, *n* (%)	3 (6.0)	0 (0.0)	**0.014**
Maximum systolic BP, mmHg, mean (SD)	207.6 (28.9)	188.3 (39.7)	**<0.001**	ASA-PS Grade, median (IQR)	3 (1)	2 (0)	**<0.001**
Maximum diastolic BP, mmHg, median (IQR)	120.7 (20.0)	98.5 (20.0)	**<0.001**	NYHA functional classification, median (IQR)	2 (0)	1 (0)	**<0.001**
Maximum mean BP, mmHg, median (IQR)	147.1 (22.1)	119.5 (35.5)	**<0.001**				

Classic triad: headache, diaphoresis, and palpitation; MEN, multiple endocrine neoplasia; VHL, von Hippel-Lindau syndrome; BP, blood pressure; HR, heart rate; MODS, multiple organ dysfunction syndrome; Renal damage: abnormal renal function test and/or urinalysis due to renal damage; Retinal damage: abnormal result in fundus examination; Hepatic damage: abnormal liver function test or PT and/or APTT due to impaired hepatic function; ASA-PS, American Society of Anesthesiologists physical status; NYHA, New York Heart Association; Other endocrine tumors: Thyroid tumors, pancreatic endocrine tumors, and pituitary endocrine tumors. Bolding means statistically significant.

Twenty-four-hour urine catecholamines are clinically considered relevant to maximum HR and BP (22). Collinearity analysis also confirmed this association. Twenty-four-hour urine catecholamines were excluded in the final binary logistic regression for this collinearity and their relatively poor predictive strength compared to maximum HR and systolic BP. Malignancy was excluded for it was not easily determinable before surgery and thus not an ideal predictive factor. All other variables that were significant in the previous analysis failed to show significance in binary logistic regressions and were excluded from the final analysis. Multivariable logistic regression analysis was then conducted. Maximum resting HR ≥115 beats/min (OR 10.05, 95% CI 3.71–27.20), maximum resting systolic BP ≥180 mmHg (OR 7.17, 95% CI 2.22–23.23), blood glucose ≥8.0 mmol/L (OR 6.52, 95% CI 2.25–18.86), ≥3 symptoms and signs (OR 6.05, 95% CI 1.86–19.64), and onset age ≤40 years (OR 3.74, 95% CI 1.37–10.20) were the independent significant predictors of CICMPP (**Table 3**).

**Table 3:** Multivariable analysis of clinical predictors for CICMPP in PPGL patients.

Covariate	OR	95% CI	*p*
Maximum HR ≥115 beats/min	10.05	3.71–27.20	**0.000**
Maximum systolic BP ≥180 mmHg	7.17	2.22–23.23	**0.001**
Blood glucose ≥ 8.0 mmol/L	6.52	2.25–18.86	**0.001**
Number of symptoms and signs ≥3	6.05	1.86–19.64	**0.003**
Onset age ≤ 40 years	3.74	1.37–10.20	**0.010**

Bolding means statistically significant.

In the 50 patients diagnosed as CICMPP, 20 (40%) were Takotsubo cardiomyopathy, 2 (4%) were inverted Takotsubo cardiomyopathy, 13 (26%) were hypertrophic cardiomyopathy, 10 (20%) were dilated cardiomyopathy, and 5 (10%) were myocarditis. Further analyses between patients with Takotsubo cardiomyopathy and other types of CICMPP were performed. Patients with Takotsubo cardiomyopathy had a shorter stature and required higher doses of alpha blocker (phenoxybenzamine). Also, more of these patients were female, complained of chest pain, and had extra-adrenal tumors. Height was excluded in the multivariable analysis for its obvious association with sex. Dosage of alpha blocker was excluded for it failed to yield a positive result in logistic regression. Upon multivariable analysis, female sex (OR 5.06, 95% CI 1.19–21.54), complaint of chest pain (OR 5.84, 95% CI 1.27–26.90), and extra-adrenal tumor (OR 8.64, 95% CI 1.82–40.94) were independent significant predictors of Takotsubo cardiomyopathy in CICMPP patients (**Table 4**).

**Table 4:** Univariable and multivariable analysis of clinical predictors for Takotsubo cardiomyopathy.

Variable	Univariable analysis			Multivariable analysis	
	Takotsubo cardiomyopathy (*n* = 20)	Other cardiomyopathies (*n* = 30)	*p*	OR (95%CI)	*p*
Female sex, *n* (%)	14 (70.0)	12 (40.0)	**0.048**	5.059 (1.188–21.544)	**0.028**
Height, cm, mean (SD)	160.0 (10.9)	167.4 (15.5)	**0.006**		
Chest pain, *n* (%)	10 (50.0)	7 (23.3)	0.071	5.838 (1.267–26.895)	**0.024**
Extra-adrenal tumor, *n* (%)	11 (55.0)	6 (20.0)	**0.010**	8.639 (1.823–40.942)	**0.007**
Dosage of alpha blocker, mg, median (IQR)	30 (37.5)	60 (37.5)	**0.012**		

Bolding means statistically significant.

## Discussion

In the present study we found five independent significant predictive factors for CICMPP: maximum resting HR ≥115 beats/min, maximum resting systolic BP ≥180 mmHg, blood glucose ≥8.0 mmol/L, number of symptoms and signs ≥3 (headache, diaphoresis, palpitation, hypertension, hypotension, chest pain, dyspnea, impaired tip perfusion, syncope, and nausea and vomiting), and onset age ≤40 years. This is to our knowledge the first time that independent predictors of CICMPP are identified in PPGL patients. Giavarini et al. and Zelinka et al. have also investigated into the possible relationships of different clinical factors with CICMPP in their retrospective studies (13, 17). Yet, due to the limited sample size, they have only found tendencies in similar aspects instead of predictors of statistical significance. CICMPP is a not so common cardiac complication in a rare disease. In this study, we identified independent predictors of CICMPP and Takotsubo cardiomyopathy for the first time, which can mostly be easily evaluated during an outpatient visit. In clinical contexts, doctors can identify these risk factors in their PPGL patients and promptly refer them for further cardiac evaluation and thorough medical preparation before surgery, restoring cardiac function in reversible cases.

We found the number of symptoms and signs rather than the classic triad as an independent predictor of CICMPP. The classic triad of PPGL is a specific but not very sensitive presentation, being reported in 17% of PPGL patients (22). Interestingly, another common symptom in PPGL, anxiety was not reported in our hospital records. This may be due to cultural reasons, since Chinese people might be more reluctant to talk about their emotions and feelings. The inclusion of other symptoms and signs like hypertension, which is more prevalent, can draw a more comprehensive picture (23). The presence of symptoms indicating cardiac involvement consisting of chest pain, dyspnea, and syncope are also highly indicative in identifying CICMPP. Catecholamine promotes increased insulin resistance, compromises insulin secretion, and decreases glucose uptake (24, 25). Up to 50% of PPGL patients have glucose intolerance, while most of their blood glucose level could return to normal after resection of their tumors (26). Twenty-four-hour urine catecholamines have, in theory, reliable relationships with CICMPP. However, this test is usually prescribed once or several times instead of daily. The day-to-day fluctuation of catecholamine secretion makes it hard for us to catch the peak level, whereas BP and HR can be monitored every day and has good correlation with catecholamine secretion levels. We also found that CICMPP patients are significantly younger. Hassan et al. reported similar findings in 2016, investigating differences between idiopathic and PPGL Takotsubo cardiomyopathy and found an age difference of 19 years (19).

We further analyzed the different types of cardiomyopathies and found that 20 (40.0%) of the 50 CICMPP patients were with Takotsubo cardiomyopathy. This result is supported by Giavarini et al. and Batisse-Lignierin et al. in their studies, reporting 6 out of 15 (40.0%) and 49 out of 145 (33.7%) Takotsubo cardiomyopathies, respectively (9, 13). We showed that independent predictors of Takotsubo cardiomyopathy in CICMPP are female sex, complaint of chest pain, and extra-adrenal tumors. Hassan et al. reported similar findings comparing exogenous and endogenous catecholamine-triggered Takotsubo syndromes in 2020, showing that Takotsubo-PPGL patients were predominantly female and that the most common clinical presentation was chest pain (27). The study of Agarwal et al. reviewing 38 cases of Takotsubo-PPGL cases also reported a high female ratio (70%) (6). Different types of cardiomyopathy require different medical management strategy prior to surgery (18). Takotsubo cardiomyopathy has a higher rate of left ventricular recovery before surgery compared to other cardiomyopathies (69.4% vs. 40.8%) (9). Identifying these patients and prompt medical preparation may lead to a more uneventful operation and better prognosis afterwards.

One limitation to this study is that this is a single-center retrospective study with limited number of cases. Further multicenter prospective study could achieve a larger sample size and may better elucidate the relationships between other risk factors and CICMPP.

In conclusion, the results of the present study indicate that maximum resting HR ≥115 beats/min, maximum resting systolic BP ≥180 mmHg, blood glucose ≥8.0 mmol/L, number of symptoms and signs ≥3, and onset age ≤40 years are independent predictors of CICMPP. Female sex, complaint of chest pain, and extra-adrenal tumors are the independent predictors of Takotsubo cardiomyopathy in CICMPP patients.

## Data Availability Statement

The datasets presented in this study can be found in online repositories. The names of the repository/repositories and accession number(s) can be found at: DOI: 10.6084/m9.figshare.18318575; Figshare.

## Ethics Statement

The studies involving human participants were reviewed and approved by the Institutional Review Board (IRB) of Peking Union Medical College Hospital (PUMCH). Written informed consent for participation was not required for this study in accordance with the national legislation and the institutional requirements.

## Author Contributions

YW collected the data and wrote the article. XY did the statistical analysis of the data. YH supervised the project and proofread the article. All authors contributed to the article and approved the submitted version.

## Conflict of Interest

The authors declare that the research was conducted in the absence of any commercial or financial relationships that could be construed as a potential conflict of interest.

## Publisher’s Note

All claims expressed in this article are solely those of the authors and do not necessarily represent those of their affiliated organizations, or those of the publisher, the editors and the reviewers. Any product that may be evaluated in this article, or claim that may be made by its manufacturer, is not guaranteed or endorsed by the publisher.

## Abbreviations

PPGL, pheochromocytoma and paraganglioma; CICMPP, catecholamine-induced cardiomyopathy in pheochromocytoma and paraganglioma.
