# Supplementary material for: Predictive Factors for Catecholamine-Induced Cardiomyopathy in Patients with Pheochromocytoma and Paraganglioma
Source: Front Endocrinol (Lausanne). 2022 Mar 8;13:853878. doi: 10.3389/fendo.2022.853878 (PMC8959126; doi:10.3389/fendo.2022.853878)
Supplement: Supplementary file 2 [file DataSheet_2.docx]

**Supplementary Materials**

**Supplementary Figure 1.** Receiver operating characteristic (ROC) curve of the following variables: number of symptoms and signs, maximum systolic BP, blood glucose, and maximum HR.

**Supplementary Figure 2.** Receiver operating characteristic (ROC) curve of onset age. Onset age is inversely associated with CICMPP. Thus, its ROC analysis is conducted separately.
